# Evaluation of Commercial Carob Syrups (*Ceratonia siliqua* L.) in Randomized Controlled Trials: Effects on Lipid, Glycaemic, and Anthropometric Parameters

**DOI:** 10.3390/foods14213676

**Published:** 2025-10-28

**Authors:** David Planes-Muñoz, María de los Ángeles Rosell, Carmen Frontela-Saseta, Rubén López-Nicolás

**Affiliations:** Food Science and Human Nutrition Department, Faculty of Veterinary, Regional Campus of International Excellence “Campus Mare Nostrum”, Campus de Espinardo, University of Murcia, 30100 Murcia, Spain; david.planes@um.es (D.P.-M.); carmenfr@um.es (C.F.-S.); rubenln@um.es (R.L.-N.)

**Keywords:** D-pinitol, commercial carob syrup, glycaemic index, cholesterol metabolism, metabolic syndrome

## Abstract

Background: Metabolic disorders such as diabetes and dyslipidaemia are intricately connected to dietary habits. This has resulted in an increasing interest in functional foods that may offer benefits for glycaemic and lipid regulation. This study aims to evaluate the effects of two commercial carob syrups on postprandial glycaemic response, serum lipid profile, and anthropometric measurements in healthy adults. Methods: The research comprised two complementary randomized and controlled trials: (i) a glycaemic test involving 20 volunteers and (ii) a six-week intervention that assessed serum cholesterol levels and body composition in a cohort of 72 participants. Volunteers were between the ages of 18 and 65 years in both studies. Results: Both syrups displayed statistical significance in low glycaemic indices (GI = 56.04 ± 13.75, and 60.46 ± 26.92%) and an attenuation of the postprandial glucose response by 16% compared to glucose control (*p* < 0.05). Furthermore, daily consumption of carob syrup was associated with a statistically significant reduction, *p* < 0.05, in total cholesterol and waist circumference: −14.68 ± 25.60 mg/dL and −3.58 ± 1.79 cm, respectively. These effects are attributed to the bioactive compounds naturally present in carob syrup, particularly D-pinitol and polyphenols, which may play a role in modulating insulin sensitivity and lipid metabolism. Conclusions: These findings indicate that carob syrups may serve as promising functional ingredients with a reduced glycaemic impact and potential cardiometabolic benefits. However, they should be interpreted as preliminary evidence. The novelty of the present study lies mainly in the use of commercially available syrups in a healthy cohort, where effects on lipids and anthropometry were modest but consistent.

## 1. Introduction

Metabolic diseases, including Type 2 diabetes and cardiovascular disorders, constitute two of the four principal non-communicable diseases (NCDs), accounting for an 18% risk of premature mortality from one of these conditions prior to reaching the age of 70 [1]. In 2022, approximately 828 million adults—aged 18 and older—were diagnosed with diabetes, reflecting an increase of 630 million cases since the 1990s [2]. This substantial rise has been observed in low- and middle-income countries, with particular emphasis on regions such as Southeast Asia, the Middle East, North Africa, and Latin America [3]. Diabetes is defined as a chronic condition that arises when the pancreas fails to produce sufficient insulin or when the body is unable to effectively use the insulin it does produce [4]. The primary cause of mortality associated with diabetes is vascular complications, particularly cardiovascular diseases. These conditions are closely linked to dyslipidaemia and hepatic steatosis, which are characterized by abnormal levels of lipoproteins in the bloodstream and fat accumulation within the liver. This association underscores the significant pathological mechanisms that underlies these diseases [5].

Lifestyle interventions, especially diet and exercise, serve as critical determinants in the prevention of metabolic diseases. Research has indicated that various dietary patterns —including the Western-style diet, vegetarian diet, and Mediterranean diet—interact with the composition and functionality of the gut microbiota [6]. Furthermore, alterations in gut bacterial populations, referred to as dysbiosis, have been found to influence metabolic disorders, including diabetes and dyslipidaemia [7]. In this context, there has been a growing scientific interest in the role of functional foods and ingredients in the modulation of metabolic parameters, particularly those capable of improving lipid profiles and attenuating postprandial glycaemic responses.

*Ceratonia siliqua* L., commonly known as carob, belongs to the genus *Ceratonia* within the family *Fabaceae* and has traditionally served multiple purposes, including providing sustenance for livestock, or facilitating winemaking [8]. In recent decades, carob pods have emerged as a significant food ingredient, particularly within health-oriented markets, owing to their nutritional profile and associated health benefits in the prevention of various chronic diseases [9]. Bakery and pastry products, along with carob-based milk beverages, represent the primary applications in the development of innovative and functional food products derived from carob, given its potential to serve as a substitute for cocoa [10]. The utilization of carob pods in these offerings may be achieved through various forms, including flour, powder, and syrup [11].

The nutritional carob syrup profile indicates the presence of crude fibre (3.3%), low protein content (1.4%), alongside a significant sugar content (63.88%), predominantly composed of glucose, fructose, and sucrose, with no detectable fats [12]. Among the identified minerals, potassium (K), phosphorus (P), and calcium (Ca) are present in the highest concentration. In terms of bioactive components, carob syrup exhibits a total phenolic compound (TPC) concentration ranging from 110.13 to 323.46 mg of gallic acid equivalents (GAE) per 100 g. This profile encompasses various phenolic compounds, including hydroxybenzoic acid, hydroxycinnamic acid, flavanols, flavanones, flavones, and flavonols, with gallic acid identified as the most prevalent phenolic compound [13,14]. It is essential to recognize that the concentration of these compounds may vary based on factors such as cultivar origin, seasonal conditions, and environmental factors, among others [15]. Additionally, compared to other plant-based sources or legumes, such as lentils, chickpeas or soybeans, carob syrup is acknowledged as a particularly rich source of D-pinitol [16,17]. D-pinitol is a methylated derivative of inositol and it is the most abundant inositol ether in plants. In fact, D-pinitol is a ubiquitous cyclitol in the Leguminosae and Pinaceae families [18]. Notably, higher D-pinitol concentrations have been documented in the syrup, compared to the flesh and seeds of the carob [17]. The syrup production process, which encompasses concentration and selective extraction utilizing water—in which D-pinitol is highly soluble—results in a final product that contains a comparatively elevated concentration of D-pinitol [19].

D-pinitol, a methylated cyclitol, has the potential to exert beneficial effects on glycaemic control and lipid metabolism. In fact, D-pinitol serves as a protector of the endocrine pancreas and demonstrates insulin-mimetic activity, which is advantageous for the treatment and prevention of conditions associated with insulin resistance [20,21]. The mechanism functions positively in the regulation of insulin-mediated glucose uptake in the liver by facilitating the translocation and activation of the PI3K/Akt signalling pathway [22]. Likewise, D-pinitol has demonstrated potential hepatoprotective effects through its ability to reduce the activity of liver enzymes associated with hypercholesterolemia and fat accumulation, including HMG-CoA reductase (HMGR), acyl-CoA-cholesterol acyltransferase (ACAT), and cytochrome P4502E1 (CYP2E1) [23]. Although these findings are promising, most evidence comes from in vitro or animal studies, and additional research is needed to clarify the precise mechanisms in humans.

Numerous studies conducted in vitro and in vivo highlight the therapeutic potential of carob syrups for metabolic health [24,25]. However, there exists a significant lack of understanding of its effects in human trials, particularly involving commercially available syrups. Consequently, it is essential to examine the effects of carob syrup consumption on glycaemic, lipid, and anthropometric parameters in volunteers. Given this framework, the objective of this research is to assess the hypoglycaemic and hypocholesterolaemic effects of commercial carob syrups in normoglycaemic and normolipidemic volunteers through a randomized controlled intervention with glycaemic response testing, serum lipid profiling, and anthropometric measurements.

## 2. Materials and Methods

### 2.1. Carob Syrup

Two commercial carob syrups (Ceratonia^+^), manufactured by Gregorio Martínez-Fortún SL (Murcia, Spain), were selected to investigate their metabolic effects in healthy adult volunteers: (i) Ceratonia^+^ Black Essence^®^, and (ii) Ceratonia^+^ Gold Essence^®^ (Ceratonia^+^). Ceratonia^+^ Black Essence^®^, produced by direct aqueous extraction of whole carob pods, is a dark-brown coloured syrup characterized by its dense texture, intense aroma, and strong flavour. Ceratonia^+^ Gold Essence^®^, derived from the refined carob pods, extracted with water, and without chemical substances, exhibits a yellow-gold appearance and a milder sensory profile. Table 1 includes the composition and bioactive compounds of the carob syrups provided by the manufacturer and used as reported.

### 2.2. Study Design

**Figure 1:**
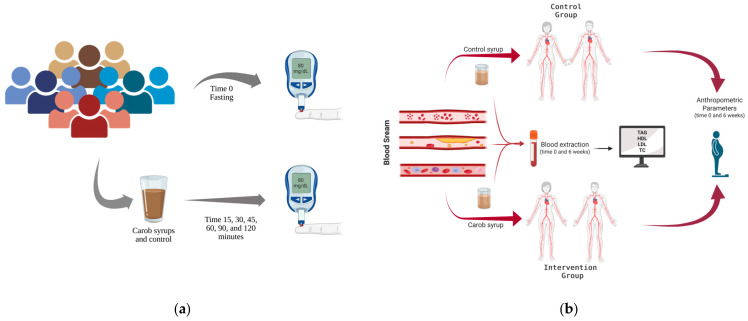
Study design: (**a**) glycaemic evaluation; (**b**) cholesterol assessment.

The research comprised two complementary randomized controlled trials aimed at evaluating the metabolic effects of commercial carob syrups (Figure 1):

(i) Glycaemic index (GI);
(ii) Serum cholesterol levels and anthropometric measurements.

**Figure 2:**
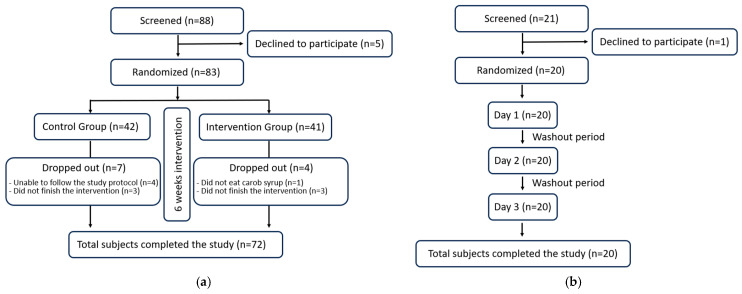
CONSORT 2025 flow diagram: (**a**) for the cholesterol and anthropometric intervention trial; (**b**) for the glycaemic index. The figure follows CONSORT 2025 guidelines for randomized controlled trials.

To ensure that the trial report is clear and transparent, the CONSORT 2025 list [26] was checked for the different items, and a CONSORT flow diagram is included (Figure 2). The Bioethics Committee of the University of Murcia granted approval for this study in accordance with the ethical principles established in the current Declaration of Helsinki of the World Medical Association, bearing the codes 2474/2019 and 4755/2023 for GI and serum cholesterol levels studies, respectively. To ensure informed decision-making, each participant provided their written and sign informed consent prior to engaging in this study. Recruitment of volunteers was conducted through university and city posters, word-of-mouth, personal contacts, and email. The participants did not receive any form of monetary compensation for their involvement in this study. Furthermore, to maintain transparency and accountability, the study protocol of these randomized trials was registered on the Open Science Framework (osf.io/7ar5m/; accessed on 26 September 2025 ).

#### 2.2.1. Glycaemic Index (GI)

The GI was assessed by monitoring blood glucose levels in a cohort of 20 normoglycaemic volunteers following the ingestion of each food sample, specifically the Black and Gold^®^ product manufactured by Ceratonia^+^, according to the procedure described by Brouns et al. [27]. Study participants were (a) adults aged 18 to 65, (b) non-smokers, with (c) a body mass index (BMI) below 29.9 kg/m^2^. Exclusion criteria included severe chronic diseases (e.g., coronary heart disease, diabetes mellitus, kidney, liver, or endocrine disorders), pregnancy or lactation, and use of medications affecting glucose metabolism, including hormonal contraceptives. This study was conducted in the morning, after an overnight fasting period of 10 to 12 h for the subjects. The study design is presented as a flow diagram in Figure 1a.

Blood glucose measurements were acquired through finger pricks utilizing sterile, single-use lancets Verifine 23G/1.8mm (Promisemed Hangzhou Meditech Co., Ltd, Hangzhou, China). Subsequently, blood glucose levels were assessed employing the MultiCareIn^®^ device (Biomedical Systems Corporation, Maryland Heights, MO, USA), in accordance with the manufacturer’s specifications. The GI test was conducted using a randomized crossover design, wherein each participant evaluated the three products (two carob syrups and glucose control) on separate days. A minimum 7-day washout period was implemented between sessions to prevent potential carry-over effects. Fasting blood glucose levels were recorded at baseline, 15, 30, 45, 60, 90, and 120 min following the ingestion of the test products containing 15.7 g of carob syrup diluted in 250 mL of water, which contains 10.4 g of carbohydrates. It was repeated on three different days for each sample. The first glucose sample was taken exactly 15 min after the first sip of the drink. A glucose solution, containing an equivalent amount of carbohydrates as the syrups, excluding inositol, was utilized as the control and was also diluted in 250 mL of water.

Results are presented as the average blood glucose values, mg of glucose per dL of blood over time for each syrup analysed. In addition, the area under the glucose response curve (AUC) was calculated for both the syrups and the control solution. The GI for each test meal consumed was expressed as the percentage of the mean AUC with respect to the control glucose by the same subject, as follows. All data results are reported as a mean ± the standard deviation (SD), or mean ± the standard error of the mean (SEM).

#### 2.2.2. Evaluation of Serum Cholesterol Levels

The serum cholesterol levels were assessed through the analysis of biochemical and anthropometric parameters involving 72 normolipidemic participants. Participants were selected according to the same inclusion and exclusion criteria established for the determination of the glycaemic index. The study design is presented as a flow diagram in Figure 1b. The participants were divided into two distinct groups:

(i) Control group (CG), including 35 participants who consumed the same quantity of a syrup product that maintained an identical sugar concentration yet did not contain carob or D-pinitol and matched the colour and consistency of the carob syrup.
(ii) Intervention group (IG), including 37 participants who ingested Ceratonia^+^ Black Essence^®^ carob syrup.

A nutritionist interviewed participants at the beginning and end of this study to assess eating habits and provide standardized Mediterranean diet and lifestyle recommendations. The prescribed diets were formulated to be isocaloric. For each participant, basal metabolic rate (BMR) was calculated using the Harris–Benedict equation. Total energy expenditure (TEE) was determined by applying the appropriate physical activity factor and a thermic effect of food (TEF) set at 10%. The diets were nutritionally balanced, providing approximately 55% of total energy from carbohydrates, 15% from protein, and 30% from fat. All meal plans adhered to Mediterranean diet principles, prioritizing the intake of fruits, vegetables, whole grains, olive oil, and other traditional Mediterranean foods.

Participants received the syrup only at the beginning of this study and were instructed to consume 10 g of their respective syrups daily, six days a week, over a period of six weeks, directly, without dilution, with optional suggestions for intake such as on toast or with yogurt. Participants took 10 g per day of carob syrup, corresponding to an intake of approximately 1.0 g of D-pinitol. This estimate is derived from previously reported concentrations of 60–100 mg/g of D-pinitol in carob syrup, which represent the reference range associated with scientifically observed health effects [17,28,29]. A total of eleven volunteers withdrew from this study for personal reasons, including seven from the CG, and four from the IG.

Each participant attended two scheduled visits to our facilities to undergo the necessary measurements for this study: one visit occurred at the commencement and the second at the conclusion of the six-week period. The measurements were categorized as follows:

(i) Biochemical parameters, including total cholesterol (TC), high-density lipoprotein (HDL), low-density lipoprotein (LDL), and triglycerides (TAG).
(ii) Anthropometric parameters, including weight, height, waist and hip circumferences, percentage of muscle mass, and fat mass.

Blood participant samples were processed at Eurofins Munuera SL (Murcia, Spain) to measure biochemical parameters. For each parameter analysed, the increases in values were calculated and, subsequently, organized by group (intervention and control), with the results presented alongside their respective SEM. In the cholesterol and anthropometric study, the changes in each variable were determined by calculating the difference between post-intervention and pre-intervention values and expressed as means ± SD, or mean ± confidence interval (CI) with a 95% confidence level, expressed as lower and upper limits.

### 2.3. Data and Statistical Analyses

Prior to data collection, sample size was calculated using the R package pwr (version 4.5.1) to ensure adequate sample sizes for both randomized trials. For the GI study, the pwr.anova.test function was used with *k* = 3, effect size (*η^2^*) = 0.44, significance level = 0.05, and statistical power = 0.8, resulting in a minimum of 17 participants required (*n* = 17.36). For the cholesterol and anthropometric intervention, the pwr.t.test function was used, with an effect size (*d*) = 0.50, significance level = 0.05, and statistical power = 0.8, yielding 33 participants per groups as the minimum sample size (*n* = 33.01). Both studies therefore achieved sufficient statistical power to detect medium effect sizes under a 5% significance criterion. Effect size data were obtained from previous research conducted by our group.

Software R (version 4.5.1) was used to perform all statistical analyses and figure generation. For the analysis of cholesterolaemia, numerical variables, including TC, LDL, HDL, TAGs, hip circumference, waist circumference, fat mass, visceral mass, muscle mass, and weight were assessed. Incremental values for each variable and group were calculated. Assumptions of normality and homogeneity of variances were evaluated using the Shapiro–Wilk and Bartless tests, respectively. Differences in incremental means between control and intervention groups were analysed using the Student’s *t*-test for independent samples. For GI means, the Student’s *t*-test for paired samples was applied. Comparisons of glycaemic area under the curve were conducted using a one-way analysis of variance (ANOVA) for repeated measures, followed by a post-hoc Dunnett test to assess group differences. Assumptions for these analyses were verified using the Shapiro–Wilk test for normality and Mauchly’s test for sphericity. Statistical significance was set at *p* < 0.05. Data normality, homogeneity of variances, and sphericity were confirmed prior to analysis using the Shapiro–Wilk, Bartlett, and Mauchly’s sphericity tests, respectively. Missing data were excluded from analyses.

## 3. Results and Discussion

### 3.1. Glycaemic Index

**Figure 3:**
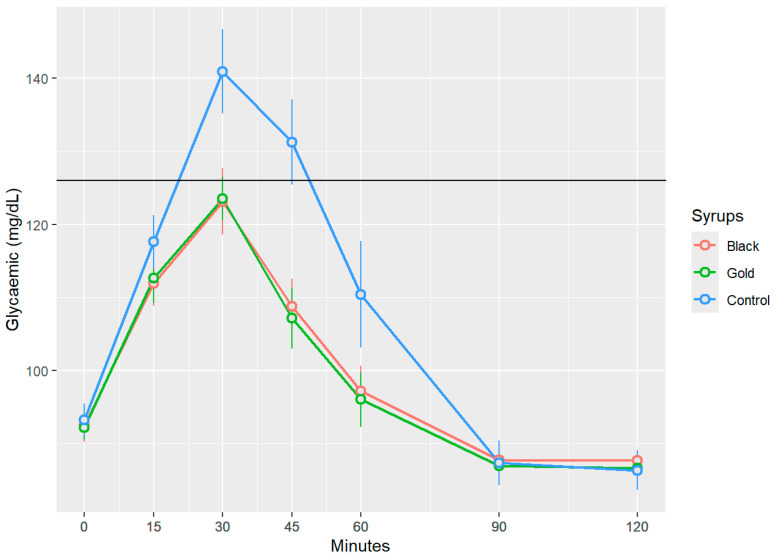
Glycaemic response to glucose and carob syrups. Blood glucose values are expressed in mg/dL over time (measured in minutes) for each analysed syrup. The data presented in this graph represent blood glucose values with baseline glucose levels subtracted, as mean (points) ± SEM (error bars). Indicates statistically significant differences between the samples at the same measured time point (*p* < 0.05).

Figure 3 presents the changes in postprandial blood glucose levels following the ingestion of the three treatments. Black and gold commercial syrups resulted in increases in blood glucose levels that exhibit a similar temporal pattern, reaching maximum glucose values, respectively, of 123.13 ± 17.41 mg/dL, and 123.53 ± 11.06 mg/dL (mean ± SD) at 30 min without surpassing the 126 mg/dL threshold and suggesting a rapid incorporation to the blood stream. In contrast, the control syrup showed significantly, *p* < 0.05, elevated peak blood glucose level at the 30-min mark, measuring approximately 140.93 ± 21.98 mg/dL (mean ± SD), thereby exceeding the established threshold of 126 mg/dL and indicating a hyperglycaemic response. Beginning at the 60 min mark, the glucose levels across all three groups began to decline progressively, approaching baseline values by the 100 and 120 min time points. Notably, no statistically significant differences (*p* > 0.05) were detected between the two syrups at any measured time point (15, 30, 45, 60, 90, and 120 min) for postprandial blood glucose.

Similar findings were reported by Navarro et al. [30]: following the oral human administration of 50 g of carob syrup, blood glucose levels progressively increased, reaching a peak at 30 min post-administrations, and remained stable for at least one hour thereafter, with maximum values of 120 mg/dL. Papakonstantinou et al.’s research [31] showed that volunteers who ingested a carob snack, containing 35 g of flour carob and 32 g of carob syrup per 100 g of snack, generated a significantly maximum peak load at the 30–45 min mark, with blood glucose value levels of 95 mg/dL, showing a similar progressive decline after the 60 min mark. Meanwhile, the glucose reference drink reached values of 120 mg/dL during the same period of time. Human studies have demonstrated that food enriched with D-pinitol derived from carob pods enhances insulin sensitivity and reduces inflammatory markers in individuals with prediabetes, indicating that D-pinitol is the compound behind this hypoglycaemic response due to its insulin-mimetic activity acting through post-receptor mechanisms [32,33]. The mechanism involves the activation of the PI3K/Akt signalling pathway, a central cascade in insulin-mediated cellular responses [34,35]. This activation promotes the translocation of the insulin-sensitive glucose transporter GLUT4 to the plasma membrane of skeletal muscle and adipose tissue, thereby facilitating glucose uptake and enhancing glycogen synthesis, ultimately leading to a significant reduction in blood glucose concentrations [22,36].

**Figure 4:**
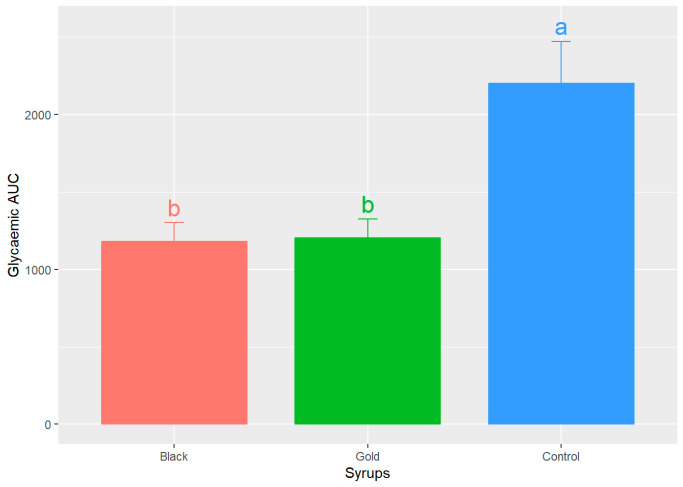
Mean values of the areas under the curve (AUC) ± SEM for each analysed syrup. Different letters (a,b) indicate significant statistical differences (*p* < 0.05).

Figure 4 illustrates the results of the area under the curve (AUC) calculations for both the control curve and the two syrups. This measurement is essential, as it indicates the actual glycaemic load of each food. The AUC values for both syrups exhibit a comparable range, with no statistically significant differences (*p* > 0.05) detected between the black and gold syrups (1179.50 ± 472.16 and 1202.00 ± 474.22, respectively) (mean ± SD), both being effective in promoting the return to baseline glucose concentrations. Nevertheless, upon comparing the study samples to the control curve, 2202.00 ± 1048.50 (mean ± SD), statistically significant differences are observed (*p* < 0.05).

Similarly, Restuccia et al.’s study [37] evaluated the GI of two carob flour-based bakery products with 20 and 40% of carob flour by calculating the AUC and obtained similar values for the bakery product with 40% of carob flour. Another research reported AUC blood glucose values for bread enriched with fine wheat bran and 10% carob seed flour that were significantly lower than those of the reference D-glucose food [38]. The AUC once again demonstrated that the participants who received the glucose control solution exhibited significantly greater and more prolonged deviations from basal glucose levels.

In addition to D-pinitol, bioactive compounds such as fibre and polyphenols have also been found to be involved in the hypoglycaemic effect of carob. These compounds, as reported by Edwards et al. [39], are linked to each other by hydrogen bonds, maintaining gut health. Despite the absence of fibre in the nutritional composition of both commercial carob syrups, polyphenols represent a significant fraction of their bioactive constituents (Table 1). Current evidence suggests that a wide range of dietary polyphenols can modulate carbohydrate metabolism through multiple mechanisms, such as inhibiting glucose transporters or modulating pathways involved in carbohydrates metabolism [40,41]. Concretely, galloyl-hexoside, the major polyphenol constituent in both commercial syrups, and characterized by the linkage of gallic acid to a hexose sugar, exerts hypoglycaemic effects from the inhibition of α-glucosidase activities. This compound functions as an inhibitor of α-glucosidase, an enzyme that facilitates the breakdown complex carbohydrates into glucose. It exhibits a competitive inhibitory mechanism by binding to both the active and non-active sites of α-glucosidase, thereby forming an inhibitor–enzyme-substrate complex. This complex effectively slows the absorption of glucose into the bloodstream, thereby mitigating postprandial spikes in blood sugar levels [42].

**Figure 5:**
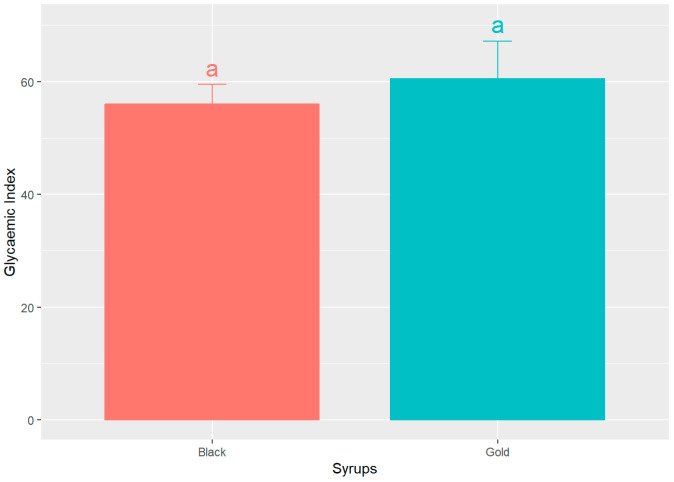
Mean glycaemic index (GI) values ± SEM, for the two analysed syrups, expressed as percentage (%). Different letters indicate significant statistical differences (*p* < 0.05).

Figure 5 shows the GI of both syrups, determined using the glucose AUC normalized as the reference as 100%. The data indicates that black and gold syrups exhibit comparable glycaemic indices, 56.04 ± 13.75% and 60.46 ± 26.92% (mean ± SD), respectively. However, the black sample displays a marginally lower value. Notably, there are no statistically significant differences (*p* > 0.05) observed between the two syrups.

These findings are consistent with the research conducted by Milek dos Santos et al. [43], who administered a carob bar containing 26 g of available carbohydrates to seven healthy volunteers, yielding a glycaemic index of 38.6%. Furthermore, despite their sweet flavour profiles, both syrups exhibit a low glycaemic index that is approximately 40% lower than that of glucose. This suggests that foods characterized by a low glycaemic index facilitate slower postprandial increases in blood glucose levels, thereby providing a gradual supply of energy to the body.

Collectively, the result obtained from the glycaemic response profiles, the AUC analyses, and GI determination strongly indicate that both commercial syrups attenuate the postprandial glycaemic response in comparison to the glucose control group, potentially indicating a reduced metabolic impact following consumption of the carob syrups. In line with our findings, several previous human trials have consistently reported a reduced glycaemic impact of diverse carob-derived products [30,31,43,44]. These converging results indicate that the hypoglycaemic effect of carob is well supported by earlier human studies. However, the present study extends this evidence by demonstrating that such effects are also reproducible with commercially available carob syrups, thereby reinforcing their applicability as functional ingredients in real dietary settings. The comparable metabolic behaviour of both commercial syrups underscores the robustness of their glycaemic modulation potential, asserting that both syrups, in spite of their intrinsic content of sugars, possess a diminished glycaemic load that glucose. Furthermore, these findings support the hypothesis that the incorporation of bioactive constituents such as D-pinitol and polyphenols may effectively modify the glycaemic index of glucose/fructose syrup, improving glucose handling and insulin responses. The consistent results observed remained unaffected by the distinct sensory characteristics of the two commercial syrups, which include variations in colour, flavour, and texture. This observation indicates that their metabolic behaviour is reproducible and is predominantly influenced by their bioactive composition, rather than by any organoleptic differences.

### 3.2. Serum Cholesterol Evaluation

#### 3.2.1. Biochemical Parameters

**Figure 6:**
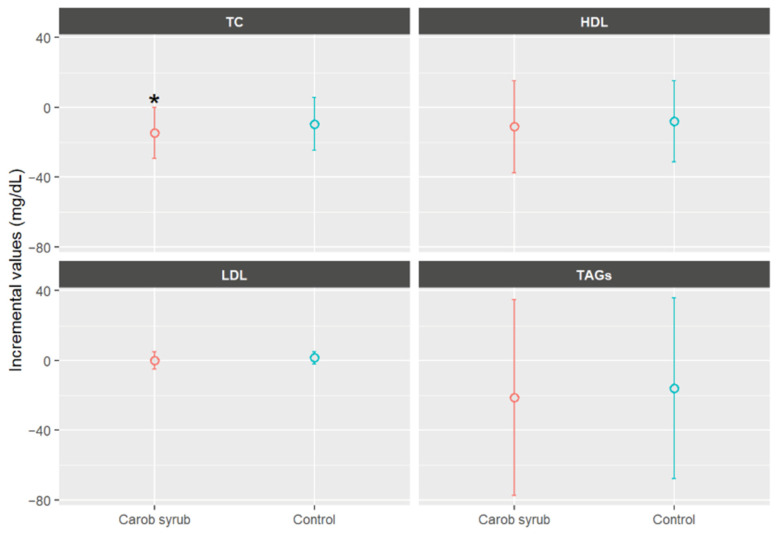
Mean increases values ± CI (95%) in biochemical parameters, expressed in mg/dL, separated by group after six weeks of study. * Indicates statistically significant differences (*p* < 0.05) between the beginning and end of this study for the indicated parameters. TC: total cholesterol; LDL: low-density lipoprotein; HDL: high-density lipoprotein; TAGs: triacylglycerides.

Figure 6 presents the biochemical mean values parameters in volunteers who consumed either the carob or the control syrup, expressed as the difference between final and baseline measurements. As can be seen, the consumption of carob syrup was associated with a statistical mean reduction in TC of −14.68 ± 25.60 mg/dL (mean ± SD), compared to a reduction of −9.57 ± 27.46 mg/dL (mean ± SD) in the control group, *p* < 0.05. Importantly, the reduction in TC observed in the carob syrup group was statistically significant when comparing baseline and final values (*p* < 0.05), an effect not observed in the control group. Regarding the detailed lipid profile, HDL cholesterol levels remained largely unchanged in both groups (−11.09 ± 25.06 and −7.91 ± 28.51 mg/dL, *p* > 0.05, mean ± SD), while LDL cholesterol exhibited a slightly greater decrease in participants receiving carob syrups (−0.14 ± 7.30 mg/dL, *p* > 0.05, mean ± SD). The TAG level was reduced by −21.44 ± 82.14 mg/dL (mean ± SD) in the carob syrup group relative to the control (−15.95 ± 30.86, *p* > 0.05, mean ± SD). Overall, the six-week intervention with carob syrup resulted in modest improvements in biochemical parameters, with all lipid markers showing greater improvements in the carob syrup group compared to the control group, although between-group differences did not reach statistical significance. The results should be approached with caution due to the modest sample size and the relatively short duration of the intervention. These limitations may have restricted statistical power to detect significant differences between the groups.

These outcomes corroborate prior evidence concerning the hypolipidemic effects of carob-derived products, which are attributed to the bioactive components naturally present in carob syrups, particularly D-pinitol, polyphenols, and soluble fractions of carob-derived carbohydrates [16]. As described previously, D-pinitol acts as an insulin-sensitizing and anti-inflammatory compound, capable of improving glucose handling and, indirectly, lipid metabolism through the modulation of the PI3K/Akt signalling pathway [32]. Choi et al. [45] conducted research indicating that the binding affinities of D-pinitol to the active site of HMG-CoA reductase exceed those of atorvastatin. HMG-CoA reductase is an enzyme that plays a vital role in the synthesis of cholesterol with the body. The superior docking ability of D-pinitol to HMG-CoA reductase significantly influences its enzymatic activity, resulting in a reduction in its activity. This interaction is beneficial in regulating cholesterol production and metabolism. However, this mechanism, based on in silico docking data, should be regarded as hypothetical. Further in vivo confirmation is necessary to validate these molecular interactions.

Quercetin represents a relevant bioactive compound that influences total cholesterol levels [46]. Research conducted by Xiao et al. [47] indicated that four specific bioactive flavonoids, including quercetin, contribute to the down-regulation of mRNA expression for sterol regulatory element-binding protein 2 (SREBP-2). This transcription factor is pivotal, as it regulates the expression of the LDL receptor and the genes associated with cholesterol synthesis. Furthermore, caffeic acids and their derivatives are associated with cholesterol synthesis through the reduction of HMG-CoA reductase and SREBP-2 activity. This indicates that caffeol compounds may enhance the activation of AMPK and facilitate lipid-lowering effects by inhibiting cholesterol biosynthesis [48].

Our findings are consistent with previous randomized clinical trials reporting lipid-lowering effects of carob-derived products, particularly in hypercholesterolemic populations. Zunft et al. [49] observed significant reductions in total and LDL cholesterol with carob pulp fibre supplementation, while Ruiz-Roso et al. [50] reported decreases of 18% in TC, 23% in LDL, and 16% in TAGs after daily intake of carob fibre in hypercholesterolemic adults. Similarly, Martínez-Rodríguez et al. [51] found a significant cholesterol-lowering effect in hypercholesterolemic subgroups consuming a polyphenol-rich carob extract incorporated into dairy products. Compared with these trials, the reductions observed in our healthy cohort were smaller in magnitude, which is likely attributable to the normolipidemic baseline of the participants and the sort of duration of the intervention. This highlights both the safety and the potential carob syrup as a preventive dietary tool in healthy populations, while suggesting that more pronounced lipid-lowering effects may be achievable in individuals with elevated baseline cholesterol levels. The findings indicates that while a balanced diet alone may support lipid profile improvement, the inclusion of carob syrup as a dietary supplement may confer additional lipid-lowering benefits.

#### 3.2.2. Anthropometric Parameters

**Figure 7:**
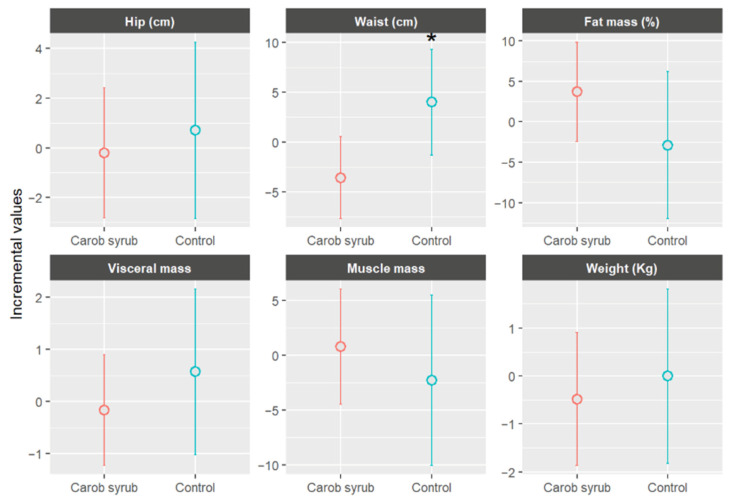
Mean increases values ± CI (95%) in anthropometric parameters, separated by group, after six weeks of study. * Indicates statistically significant differences (*p* < 0.05) between the beginning and end of this study for the indicated parameters.

Figure 7 illustrates the mean changes in anthropometric parameters, stratified by group, following six weeks of intervention. In the intervention group, which consumed carob syrup, favourable trends were observed in comparison to the control group across multiple variables. Participants in the intervention group experienced an average reduction in body weight of −0.49 ± 1.48 kg (mean ± SD) (*p* > 0.05) relative to the control. Improvements were also noted in muscle mass percentage, visceral fat, and waist and hip circumferences. Visceral fat levels showed a slight decrease in the intervention group, −0.17 ± 1.17 (mean ± SD), whereas a slight increase was recorded in the control group, +0.57 ± 1.14 (mean ± SD), (*p* > 0.05). Waist circumference was statistically reduced, *p* < 0.05, in the carob syrup group, with a mean decrease of approximately −3.58 ± 1.79 cm (mean ± SD) over the six-week periods. Hip circumference remained relatively stable, without significant statistical significance, showing a modest decline in the intervention group, −0.20 ± 3.49 cm (mean ± SD), and a slight increase in the control group, +0.70 ± 1.52 cm (mean ± SD), (*p* > 0.05). Consequently, the waist-to-hip ratio improved in the group receiving carob syrup.

The significant statistically, substantial reduction in waist circumference, alongside observed trends of decreased body weight and visceral fat, indicates a potential role of this product in the management of abdominal adiposity. Central obesity is recognized as a significant early indicator of metabolic risk, and its reduction holds clinical relevance, even in the absence of considerable weight loss. As mentioned previously, the results of this investigation align with prior clinical and observational data, indicating that compounds such as D-pinitol and polyphenols contribute to enhancements in insulin sensitivity, lipid metabolism, and the mitigation of low-grade inflammation [52,53]. These mechanisms may promote a redistribution of body fat and a reduction in central obesity, even in healthy volunteers. For instance, a 12-week randomized trial conducted with prediabetic subjects consuming a beverage enriched with D-pinitol reported notable improvements in insulin resistance, glycaemic variability, and inflammatory cytokines [33]. Notably, the non-obese participants exhibited stability in body weight, whereas the obese participants demonstrated reduction in inflammatory markers despite minimal changes in weight. Similarly, a study examining healthy individuals revealed that chronic consumption of a carob-derived inositol-enriched beverage, which included 2.2 g of inositol taken twice daily, significantly improved metabolic markers without a corresponding substantial gain in weight [54]. In contrast, control groups revealed increases in BMI.

While the reductions in weight and waist circumference observed in this study are modest, meta-analyses of dietary interventions that include polyphenol-rich foods have reported comparable effects on adiposity, such as reductions in waist circumference of 0.6 to 1.0 cm and body weight declines of approximately 0.3 to 1.0 kg over similar durations [55]. It shows that even minor shifts in anthropometric measures can correlate with biologically significant metabolic benefits, particularly when accompanied by biochemical improvements. Mechanistically, polyphenol-rich foods, including carob syrups, exert anti-adiposity effects through several pathways: modulation of gut microbiota, inhibition of adipogenesis, activation of AMPK, and PI3K/AKT signalling pathways, as well as suppression of inflammation [56,57,58]. These processes are implicated in weight and fat reduction mechanisms. Although the changes observed may be modest in magnitude, the directionally of the effect, combined with demonstrated biochemical improvements, supports the hypothesis that regular consumption of carob syrup may play a role in the early prevention of cardiometabolic alterations through a mechanism related to enhanced insulin sensitivity, reduced lipogenesis, and diminished inflammation. It is essential, however, to interpret these observations with caution due to the relatively small sample size and the short duration of the intervention, which may have constrained the ability to detect more pronounced changes in other anthropometric variables. These results support a potential beneficial effect of carob syrup on body composition and fat distribution, particularly in reducing abdominal adiposity and possibly improving cardiometabolic risk indicators. Table 2 summarizes the main outcomes observed for the tested commercial carob syrups.

Although the reduction in waist circumference observed in the intervention group was statistically significant and clinically relevant, this result should be interpreted with caution. Previous human trials with carob-derived extracts have mainly reported improvements in insulin sensitivity and inflammatory markers without marked anthropometric changes [54]. The relatively small sample size, short intervention period, and the inclusion of healthy adults may partly explain the discrepancy with prior findings. Thus, while our results suggest a potential role of carob syrup in modulating abdominal adiposity, stronger anti-adiposity claims cannot be made at this stage and require confirmation in longer-term studies with larger and metabolically at-risk populations. The possibility of measurement variability or bias cannot be excluded, and replication in larger, longer-term trials is required before firm anti-adiposity claims can be made.

## 4. Conclusions

The present study shows preliminary evidence that the regular consumption of commercial carob syrups may yield modest improvements in glycaemic responses, lipid profiles, and abdominal adiposity in healthy adults. Specifically, the syrups exhibited a low glycaemic index (GI = 56.04 ± 13.75 and 60.46 ± 26.92%), diminished postprandial glycaemic responses by 16% compared with the glucose, and contributed to modest enhancements in lipid profiles and abdominal adiposity, particularly in TC (−14.68 ± 25.60 mg/dL) and waist circumference (−3.58 ± 1.79 cm) in normolipidemic adults. These beneficial effects are attributed to the presence of D-pinitol and polyphenolic compounds, such as gallic and caffeic derivatives, and flavonoids such as quercetin, which are recognized for their ability to modulate glucose and lipid metabolism through insulin-mimetic and hypolipidemic mechanisms.

Consequently, the findings of this research indicate that carob syrups represent promising alternatives to conventional sweeteners in the formulation of functional foods designed to enhance glycaemic control and support cardiometabolic health. The results align with prior human studies showing effects of carob-based products on glycaemic control and lipid metabolism. The present study adds novelty by evaluating commercial syrups in a healthy population, but the effects observed were more modest than those reported in hypercholesterolemic or prediabetic cohorts. The demonstrated low glycaemic impact and bioactive composition of these syrups substantiate their potential application as complementary tools in the preventive nutrition and management of metabolic syndrome and related NCDs. In addition, as the syrups were provided by a sponsor that had no role in the study design, data analysis, or interpretation.

The present study has several limitations, such as the short duration of the intervention and the focus on a healthy population, which restrict the generalizability of the findings. The six-week duration and modest sample size may have limited statistical power, particularly for between-group comparisons. The inclusion of healthy participants with normal baseline lipid levels restricted the magnitude of observable effects. Future clinical trials should prioritize randomized controlled trials with longer follow-up periods (≥12 weeks) and larger sample sizes, including individuals at risk, such as those with prediabetes, metabolic syndrome or dyslipidaemia. Such studies should measure detailed glycaemic profiles (e.g., postprandial glucose or insulin), comprehensive lipid panels, and anthropometric outcomes. Additionally, the assessment of inflammatory and oxidative stress markers could provide further insights into the mechanistic effects of carob syrup consumption, enabling a more precise evaluation of its potential as a functional food for the cardiometabolic health. Finally, the potential modulation of gut microbiota by carob syrup consumption should be investigated, as it may represent an additional pathway through which these syrups exert beneficial metabolic effects, aligning with the current interest in diet-microbiome interactions and functional foods.

## Figures and Tables

**Table 1:** Composition and bioactive compounds of the carob syrups (Ceratonia^+^) used in this study.

	Value		Units
	Black Essence^®^	Gold Essence^®^	
Physico-chemical characteristics			
Brix at 20 °C	65–69	65–69	ºBrix
Density	1.35	1.35	g/mL
pH	5.5	5.5	
Sweetening power	45–50	45–50	
Nutritional composition			
Carbohydrates	66.3	66.3	%
of which sugars	58.3	58.3	%
Energy	1197.8	1197.8	kJul/100 g
	282.2	282.2	kcal/100 g
Fat	1.2	1.2	%
of which saturates	<0.1	<0.1	%
Protein	1.5	1.5	%
Salt	0.2	0.2	%
Fibre	<0.1	<0.1	%
Bioactive constituents			
No Flavonoids			
Caffeoyl 4-O-glucoside	293.1	117.9	ppm
Caffeoyl-galloyl-hexoside	80.7	36.2	ppm
Galloyl-hexoside	420.2	302.4	ppm
Flavonoids			
Procyanidin dimer	23.1	21.2	ppm
Quercetin-glucoside	27.1	-	ppm
Myricetin-glucoside	85.2	-	ppm
Myricetin deoxihexoside	131.6	-	ppm
Quercetin deoxihexoside	71.5	21.8	ppm
Total polyphenols	1132.5	499.5	ppm

**Table 2:** Summary of main findings and comparison between carob syrup products.

Parameter	Black Essence^®^	Gold Essence^®^	Glucose Control	Units	Comments
Postprandial glucose	123.13 ± 17.41	123.53 ± 11.06	140.93 ± 21.98	mg/dL	Gold and black syrups significantly reduced the glycaemic responses by 16% compared with the control
GI	56.04 ± 13.75	60.46 ± 26.92	100	%	The GI values for gold and black syrups were significantly lower than those of the control
TC	−14.68 ± 25.60	-	−9.57 ± 27.46	mg/dL	Statistically major reduction with respect to the control
Waist Circumference	−3.58 ± 1.79	-	+3.99 ± 4.28	cm	Statistically major reduction with respect to the control

Mean values ± SD for each analysed syrup. GI: glycaemic index; TC: total cholesterol.

## Data Availability

The data presented in this study are available on request from the corresponding author. Public access is restricted to protect participant privacy and comply with ethical guidelines.

## Abbreviations

The following abbreviations are used in this manuscript:

NCDs	Non-communicable diseases
K	Phosphorus
P	Potassium
Ca	Calcium
HMGR	HMG-CoA reductase
ACAT	Acyl-CoA-cholesterol acyltransferase
CYP2E1	Cytochrome P4502E1
GI	Glycaemic index
AUC	Area under curve
SD	Standard deviation
CG	Control group
IG	Intervention group
TC	Total cholesterol
HDL	High-density lipoprotein
LDL	Low-density lipoprotein
TAG	Triglycerides
BMR	Basal metabolic rate
TEE	Total energy expenditure
TEF	Thermic effect of food
SREBP-2	Sterol regulatory element-binding protein 2
BMI	Body mass index
